# Impact of Vaginal Delivery on Pelvic Floor

**DOI:** 10.1055/s-0040-1709184

**Published:** 2020-02

**Authors:** Cássia Raquel Teatin Juliato

**Affiliations:** 1 Universidade Estadual de Campinas, Campinas, SP, Brazil

Pelvic floor disorders (PFDs) include urinary incontinence (UI), overactive bladder (OAB), fecal incontinence (FI) and pelvic organ prolapse (POP).^1^ Although the pathophysiology of PFDs is multifactorial, two of the main associated factors are the gestational period and the delivery route.^2^ ^3^

The pregnancy period demands several modifications in the woman's body in order to allow fetal development and childbirth. These physiological, anatomical, biomechanical and hormonal changes alter the functioning of the pelvic floor, mainly by increasing the elasticity of structures.^4^ The increase in body weight and uterine size leads to a higher abdominal pressure that overloads pelvic floor structures^5^ and provides the occurrence of injuries.

Despite the impact of pregnancy on the pelvic floor, the main factor associated with pelvic floor injuries and PFDs is childbirth, even though no consensus on the impact of the delivery route and PFDs is available in the literature.

In our service, we evaluated a cohort of primiparous women 12-24 months after delivery and found no difference between the prevalence of UI and the delivery route.^6^ Another study followed women for five years and also found no association between UI and the delivery route, although this study had only 18% of cesarean sections, which limited the results.^7^

Some studies followed postpartum women for longer periods, one of which showed that the peak risk for stress urinary incontinence (SUI) was five years after delivery, while the peak for POP was 20 years after childbirth.^8^ This finding is especially important, because it justifies the discrepancy in the literature, since studies with less than five years (SUI) and 20 years (POP) might not reveal the magnitude of changes caused by childbirth.

Handa et al.^9^ evaluated a longitudinal cohort with 1011 women five to ten years after childbirth and observed a 2.9 times greater risk of presenting SUI (OR 2.9; 95% CI 1.5-5.5) and 5.6 times greater risk of presenting prolapse (OR 5.6; 95% CI 2.2-14.7) after vaginal delivery compared to cesarean delivery without labor. Instrumental deliveries (such as forceps) were associated with an increase in all PFDs, especially POP, with a 7.5-fold increase (OR 7.5; 95% CI 2.7-20.9). The study also demonstrated that for every 6.8 instrumental deliveries or 8.9 spontaneous vaginal deliveries, there is an increase of one case of prolapse.^9^

This same cohort of women was monitored for up to nine years, resulting in a publication in 2018^8^ that showed an accumulated 15-year incidence of 34.3% of SUI (95% CI; 29.9% -38.6%); 21.8% of OAB (95% CI; 17.8% -25.7%); 30.6% of FI (95% CI; 26.4% -34.9%) and 30.0% of POP (95% CI; 25.1% -34.9%) after the first delivery. The association between the delivery route and the PFD was significant, but especially the association between vaginal delivery and POP. In addition, this study showed that cesarean delivery was significantly associated with lower risk scores for PFD.^8^

Regarding the effect of cesarean section on PFDs, 12-year follow-up studies from the United Kingdom and New Zealand have shown an association between cesarean section and lower risk for urinary incontinence, and that POP is less frequent in women who have had cesarean section.^10^ ^11^ A systematic review study showed that cesarean sections reduce the risk for SUI from 16 to 9.8% or from 22 to 10%. The number of cesarean sections required to prevent a case of SUI was estimated at 10 to 15. However, the risk for severe SUI and urge incontinence was not different when comparing the delivery routes.^12^

The pathophysiology that explains the effect of childbirth on PFDs involves several aspects such as decreased support of pelvic organs, damage to the levator ani muscle and pudendal nerve. In relation to perineal musculature, women's pelvic floor was evaluated with a perineometer in a study. It was found that women with reduced muscle strength and at least one vaginal delivery were associated with a shorter interval for presenting PFD, compared to women who had cesarean sections. In addition, the decrease in muscle strength was associated with a higher risk for SUI, 16%; OAB, 27%: and POP, 43%.^13^ In a systematic review, a decrease in muscle strength was also confirmed in women with vaginal delivery compared to those with cesarean sections.^14^ Women with complaints of decreased strength of pelvic floor muscles had avulsion of the levator ani muscle, found after evaluation by means of translabial ultrasound, and the study conducted by our research group showed an association between avulsion and vaginal delivery.^7^ In addition, 1/3 of women with a levator ani injury have decreased muscle strength.^15^ The relationship with loss of strength is extremely important after vaginal delivery because it allows the adoption of a preventive strategy in women after delivery.

DeLancey^16^ compared the stage of POP and hiatal area by predicting that the larger the hiatal area, the greater the level of POP.^15^ This shows the importance of hiatal area and the risk for PFDs. Our research team conducted a review study in which all articles included demonstrated an increase in hiatal area evaluated by 3D ultrasound after delivery, especially after vaginal delivery.^15^ The increase in hiatal area is an important risk factor for all PFDs, and an enlarged hiatal area can be considered a risk marker for POP over time.

Injuries to the levator ani are also associated with the appearance of POP, but some injuries are not so evident immediately after delivery. Studies with 3D ultrasound show there may be injury to the levator muscle in 13% of women after a vaginal delivery,^17^ although these injuries can take years to develop into POP.

Bladder neck hypermobility is also associated with PFDs, specifically SUI. However, our review article showed no difference between bladder neck hypermobility and delivery route.^15^

PFDs seem to be closely related to vaginal delivery, especially cases of instrumental delivery. Cesarean section is associated with a reduction in the risk for PFDs, although the routine performance of this procedure does not eliminate the risk for dysfunctions because the pathophysiology is multifactorial. The pelvic floor undergoes several changes during pregnancy and, especially, postpartum. Some injuries, such as injuries to the levator ani, are not routinely diagnosed. Knowledge of the pathophysiology and diagnosis of pelvic floor injuries are crucial for actions that can recover this musculature and reduce the impact of childbirth and PFDs.
